# Use of ACE inhibitors in Serbia in 2010

**DOI:** 10.1186/2050-6511-13-S1-A3

**Published:** 2012-09-17

**Authors:** Boris Milijašević, Zdenko Tomić, Ana Sabo, Aleksandar Rašković, Momir Mikov

**Affiliations:** 1 Department of Pharmacology, Toxicology and Clinical Pharmacology, Faculty of Medicine, University of Novi Sad, 21000 Novi Sad, Serbia

## Background

Cardiovascular diseases are the most frequent cause of morbidity and mortality in many countries. That explains why medications for the treatment of cardiovascular diseases are the group of drugs with the largest consumption, and ACE inhibitors take a large part in this consumption. The aim of this study was to analyze the consumption of ACE inhibitors in Serbia in 2010 compared with Norway, a country with a developed pharmacotherapeutic practice.

## Methods

The data about the use of ACE inhibitors in Serbia were taken from the Agency for Drugs and Medical Devices of the Serbia. The data about drug consumption in Norway were taken from the official website of the Norwegian Institute of Public Health.

## Results

In Serbia, the use of drugs of first choice in the treatment of hypertension was very uneven, where the consumption of ACE inhibitors was dominant. Opposed to this condition, the consumption of the first choice antihypertensive drugs was very balanced in Norway. Total consumption of ACE inhibitors in Serbia in 2010 year was 190.4 defined daily doses per 1000 inhabitants per day (DID) and total consumption of ACE inhibitors in Norway in 2010 year was 51.7 DID. During the analyzed year the largest use of plain ACE inhibitors in Serbia was for enalapril (81.0 DID), ramipril (34.2 DID), fosinopril (24.5 DID) and cilazapril (13.1 DID) and in Norway was ramipril (27.3 DID), enalapril (11.1 DID) and lisinopril (6.2 DID). In Serbia a significant part of the consumption of ACE inhibitors consisted of more expensive drugs such as fosinopril, cilazapril and quinapril, whereas these have not been used at all in Norway during that period.

## Conclusions

In Serbia in the year 2010, ACE inhibitors and their fixed combination with diuretics are the most frequently used drugs within the group of drugs which is used for cardiovascular diseases treatment. The amount and structure of the utilized ACE inhibitors in Serbia is different in a lot of ways from the amount and structure of the utilized ACE inhibitors in Norway. Pharmacoeconomic analyses also show that large financial resources would be saved if the structure of the utilized ACE inhibitors in Serbia were more similar to the one in Norway.

